# Obstructive Sleep Apnea as an Acceleration Trigger of Cellular Senescence Processes through Telomere Shortening

**DOI:** 10.3390/ijms222212536

**Published:** 2021-11-21

**Authors:** Szymon Turkiewicz, Marta Ditmer, Marcin Sochal, Piotr Białasiewicz, Dominik Strzelecki, Agata Gabryelska

**Affiliations:** 1 Department of Sleep Medicine and Metabolic Disorders, Medical University of Lodz, 92-215 Lodz, Poland; turkiewiczszymon99@gmail.com (S.T.); marta.ditmer@stud.umed.lodz.pl (M.D.); marcin.sochal@umed.lodz.pl (M.S.); piotr.bialasiewicz@umed.lodz.pl (P.B.); 2 Department of Affective and Psychotic Disorders, Medical University of Lodz, 92-215 Lodz, Poland; dominik.strzelecki@umed.lodz.pl

**Keywords:** OSA, senescence, telomere shortening, SASP, hypoxia, inflammation

## Abstract

Obstructive sleep apnea (OSA) is chronic disorder which is characterized by recurrent pauses of breathing during sleep which leads to hypoxia and its two main pathological sequelae: oxidative stress and chronic inflammation. Both are also associated with cellular senescence. As OSA patients present with higher prevalence of age-related disorders, such as atrial hypertension or diabetes mellitus type 2, a relationship between OSA and accelerated aging is observable. Furthermore, it has been established that these OSA are associated with telomere shortening. This process in OSA is likely caused by increased oxidative DNA damage due to increased reactive oxygen species levels, DNA repair disruptions, hypoxia, chronic inflammation, and circadian clock disturbances. The aim of the review is to summarize study outcomes on changes in leukocyte telomere length (LTL) in OSA patients and describe possible molecular mechanisms which connect cellular senescence and the pathophysiology of OSA. The majority of OSA patients are characterized by LTL attrition due to oxidative stress, hypoxia and inflammation, which make a kind of positive feedback loop, and circadian clock disturbance.

## 1. Introduction

Obstructive sleep apnea (OSA) is a common chronic sleep-related breathing disorder characterized by recurrent pauses in breathing, which are caused by collapsing upper airways [1]. The prevalence of OSA in the general population reaches up to 50% in men and 23% in women, and the risk of the disorder increases with advancing age, male sex and higher body mass index (BMI) [2]. OSA is commonly associated with age-related disorders such as hypertension [3,4], cardiovascular disorders [3,4], metabolic abnormalities like obesity or type 2 diabetes mellitus [5,6], and according to some studies it is also associated with some cancers [7]. The curtail complication of OSA is intermittent hypoxia, which is a main pathophysiological mechanism leading to oxidative stress and chronic inflammation [1]. Both are implicated in cellular senescence [8].

Cellular senescence is the arrest of normal cell division among others in response to multicausal oxidative stress, which leads to the overproduction of reactive oxygen species (ROS) [8]. ROS causes DNA damage and a proinflammatory response [9]. All these changes impair intracellular processes and promote irreversible tissue remodeling [10]. Cellular senescence is likely the main reason for the development of age-related diseases which have high prevalence among OSA patents [11]. That is why the cellular senescence process is closely linked to the mechanism of intermittent hypoxia in OSA patients.

Telomeres are regions of repetitive 5′-TTAGGG-3′ sequences at the ends of chromosomes. Their main function is DNA protection by the prevention of the degradation of genes near the end of chromosome arms as a result of incomplete DNA replication. Each division of cell leads to telomere shortening, due to muted expression of telomerase, an enzyme which needs to be activated to recreate chromosome endings. After multiple divisions, telomere reduction is detected as a DNA damage and leads to cell cycle arrest [12]. Considering the above, telomere shortening is one of main mechanisms of cellular senescence [12] and in vast number of studies it was used as a marker of aging.

The aim of the review is to summarize available knowledge regarding changes in leukocyte telomere length (LTL) in OSA patients and describe possible molecular mechanisms which connect the cellular senescence to the pathophysiology of OSA.

This article is narrative review and consists of a critical examination of the literature. Papers were acquired via PubMed using the following keywords or items in indexed fields: obstructive sleep apnea and telomere. The inclusion criteria included published in peer-reviewed journals, studies in humans, reviews on the related topic and English language. The exclusion criteria were: abstracts from conferences and commentaries. In addition, further articles were chosen from the reference lists of primary articles or the purpose of the investigation of pathophysiological mechanisms. The majority of studies had a cross-sectional design.

## 2. Sleep Quality and Telomere Length

Low sleep quality is a vital problem nowadays [13] and is one of the more frequent symptoms in OSA [1]. As a consequence of apneas and hypopneas, OSA patients suffer from numerous arousals which cause sleep fragmentation and which may influence the aging process [13]. Therefore, the first step of the review is to summarize available research on the influence of sleep quality on leukocyte telomere length.

Prather et al. studied the influence of sleep duration on LTL in 245 healthy women in midlife and found that poorer subjective sleep quality was associated with shorter LTL adjusted for age, BMI, race, perceived stress, and income [14]. It is important to emphasize that in the full sample, levels of perceived stress accounted for variance in the relationship between sleep quality and LTL. However, there were no correlations between LTL and measures of time in bed sleep and sleep onset latency [14]. Nevertheless, the study gave the first evidence of a connection between sleep quality and LTL shortening.

Another study, carried out among women under 50 years old from the Nurses’ Health Study (*n* = 4117), found a positive association between sleep duration and LTL [15]. Such a relationship was not reported for women above 50 years old, which might be caused by continuously progressive telomere shortening escalating with age. On the other hand, Cribbet et al. did not find any correlations between low sleep quality, sleep duration and LTL [16].

Furthermore, Jackowska et al. reported a linear association between sleep duration and LTL in men from the Whitehall II cohort study (*n* = 434). Subjects reporting shorter sleep duration had shorter LTL adjusted for age, BMI, smoking, educational attainment, current employment, cynical hostility scores and depressive symptoms. In contrast to earlier reports, this study did not offer any significant correlation for women [17]. This could be caused by higher age in this group, as all included individuals were at least 64 years old.

Garland and colleagues studied the influence of insomnia on LTL in women with breast cancer (*n* = 70), but they found only a non-significant shortening of LTL in insomnia patients, due to low number of participants [18].

Sleep quality in all these studies was measured by the Pittsburgh Sleep Quality Index (PSQI), which is a validated questionnaire and a useful supporting tool in diagnosis of patients with various sleeping disorders, including OSA. However, it is important to remember that PSQI is mostly a subjective instrument, therefore there is a limitation to its comparison to objective molecular measures such as LTL.

## 3. OSA and Telomere Length

### 3.1. Telomere Lengthening in OSA

The available literature offers contradicting data on LTL changes in OSA patients, with only a few studies indicating that OSA is associated with LTL lengthening. One of these studies investigated LTL in children with OSA. Despite their hypothesis, they found a tendency to the lengthening of LTL in this specific group of patients (*n* = 213). Moreover, LTL was positively and independently associated with apnea-hypopnea index (AHI), which strongly suggested that OSA severity had an impact on LTL [19]. Moreover, two studies by Polonis et al. showed a J-shaped association between LTL and OSA severity, while a comparison between patients with moderate-to-severe OSA and mild OSA demonstrated longer LTL in the former group [20,21]. Additionally, in one of the studies (*n* = 210) LTL was found to be significantly longer in the moderate-to-severe OSA group than the control group independently from age, sex, BMI, hypertension, dyslipidemia, and depression [21].

### 3.2. Telomere Shortening in OSA

Many more studies report LTL shortening in OSA. Barceló et al. were the first to find shorter LTL in OSA patients (*n* = 256) compared to a healthy control group (*n* = 148) [22]. What is more, these results were adjusted for age and cardiovascular risk factors such as hypertension, obesity, smoking status, cholesterol, glucose, and uric acid. Furthermore, an investigation by Savolainen showed that individuals with OSA diagnosis (*n* = 44), who required hospitalization, had shorter LTL than patients without OSA history (*n* = 1875) [23]. Nonetheless, it has to be pointed out that subjects were allocated to the groups based only on the OSA diagnosis in their medical history, without polysomnography examination (PSG) data. Due to the lack of PSG data, more detailed analysis, for example between OSA severity and LTL, was not possible in this study. Furthermore, Kwon et al. suggested that shorter LTL might contribute to the association between reduced sleep stability and OSA severity through the stress of chronic sleep fragmentation or invariant sympathetic activity by respiratory chemoreflex activation. Their study was based on cardiopulmonary coupling analysis (sleep quality) and its main limitation was the study group (*n* = 381), which consisted only of healthy individuals, allowing for limited interpretation of the results. They found an association between LTL and sleep stability (assessed by cardiopulmonary coupling) in subjects with AHI ≥ 15, elevated narrow-band low frequency coupling (marker of periodic breathing or sleep fragmentation) was predicted to increase by 0.008% during sleep as LTL was shortened by 0.1. Additionally, in the same subjects, the marker of stable sleep (evaluated by high-frequency coupling band) decreased [24].

Another report showed that LTL shortening was significantly correlated with AHI and the oxygen desaturation index (ODI) after correction for age. It should be noted that the study included only men (*n* = 161). What is more, after division into groups according to OSA severity, the authors observed significant differences in LTL between them. A comparison of non-OSA and severe OSA groups showed that the latter had shorter LTL than former. Subsequently, the authors divided the participants into three groups by ODI rate, into low, middle, and high ODI tertiles, and observed significantly shorter LTL in the high ODI group compared to low and middle ODI tertiles [25]. These results indicate the influence of severity of OSA through hypoxia and oxidative stress on telomere length. Additionally, the authors found a negative correlation between LTL and carotid-femoral pulse wave velocity, which is used as a surrogate marker of arterial stiffness (arterial aging), possibly suggesting LTL shortening as one of the development and progression mechanisms of OSA comorbidities [25].

Kim et al. focused on the association between oxidative stress and LTL in OSA patients. They found that OSA patients (*n* = 43) had higher plasma levels of hydrogen peroxide than controls (*n* = 34), which was positively correlated with the severity of OSA (assessed by the RDI–respiratory disturbance index). Additionally, this study revealed that OSA patients had shorter LTL than healthy individuals independently from BMI and age. This study was the first to offer a foundation for oxidative stress as one of the LTL shortening mechanisms in OSA patients [26]. Moreover, moderate-to-severe OSA patients with shorter LTL (*n* = 24) had about 6 times higher risk of occurrence of brain white matter abnormalities than patients with longer LTL and the same OSA severity (*n* = 36). These findings emphasize the clinical importance of early identification and treatment of OSA for brain white matter changes in elderly people [27]. Another study found that African American women with high risk of OSA have shorter LTL compared to those with low risk in a total group of 184 women. These differences were not observed in men [28]. Several studies have shown an association between LTL and PSG parameters other than OSA severity. It has been observed that LTL was significantly shorter in OSA patients (*n* = 315) compared to controls (*n* = 613) and LTL was negatively correlated with AHI, RDI, ODI and wake time after sleep onset. What is more, results revealed positive correlation between LTL and sleep efficiency, total sleep time, basal, minimum as well as maximum oxygen saturation [29]. Furthermore, study by Caroll et al. (*n* = 672) confirmed that OSA patients has shorter LTL. Collaterally, the authors found that individuals with high arousal frequency had greater LTL attrition over the prior decade, suggesting sleep fragmentation as a possible mechanism of LTL shortening [30]. In a recent prospective, an observational study by Pinilla et al. (*n* = 599) evaluated the hallmarks of ageing in OSA patients, dividing them in groups younger and older than 50 years old. A relationship has been found between the AHI, the arousal index, time during the night spent with an oxygen saturation less than 90%, and the following hallmarks: alteration of cellular communication (assessed by serum C-reactive protein concentration), deregulation of nutrient sensing (through insulin resistance), mitochondrial dysfunction (leukocyte mitochondrial DNA copy number), and genomic instability (urinary 8-hydroxy-2-deoxyguanosine concentration) [31]. Interestingly, no such link was found between the aforementioned PSG variables and telomere attrition evaluated by LTL. Based on the results of the study, it can be argued that OSA in individuals below 50 years old is associated with an increase in specific hallmarks of aging irrespective of confounding factors [31].

Not much is known on the effect of OSA treatment on the LTL. One of the reports evaluated the usage of a mandibular advancement device in OSA individuals on markers of aging like LTL and sirtuin 1 (SIRT1), which is a signaling protein involved in metabolic regulation. It has been found that, after 3 months of the treatment, participants had longer LTL and higher levels of SIRT1 compared to the baseline results, suggesting that successful treatment of OSA can inhibit accelerated aging [32]. Similar results have been reported by Khalyfa et al., who also found increased SIRT1 expression after 12 months of continuous positive airway pressure (CPAP) treatment in OSA patients [33]. Only the last two studies had longitudinal designs.

## 4. Mechanism of OSA Influence of Cellular Senescence

The previously described research points to three possible mechanisms of LTL shortening in OSA: oxidative stress, inflammation, and hypoxia.

### 4.1. Oxidative Stress and Telomere Length

Oxidative stress is the state of increased level of ROS in cells. One of the main sources of ROS in OSA patient cells is intermittent hypoxia [34]. Numerous studies of human tissues, mouse models and cell cultures provided evidence that oxidative stress is associated with accelerated LTL shortening, as was reviewed by Barnes et al. [35]. However, only one study evaluated and revealed the association between oxidative stress and LTL in OSA patients [26].

**Figure 1:**
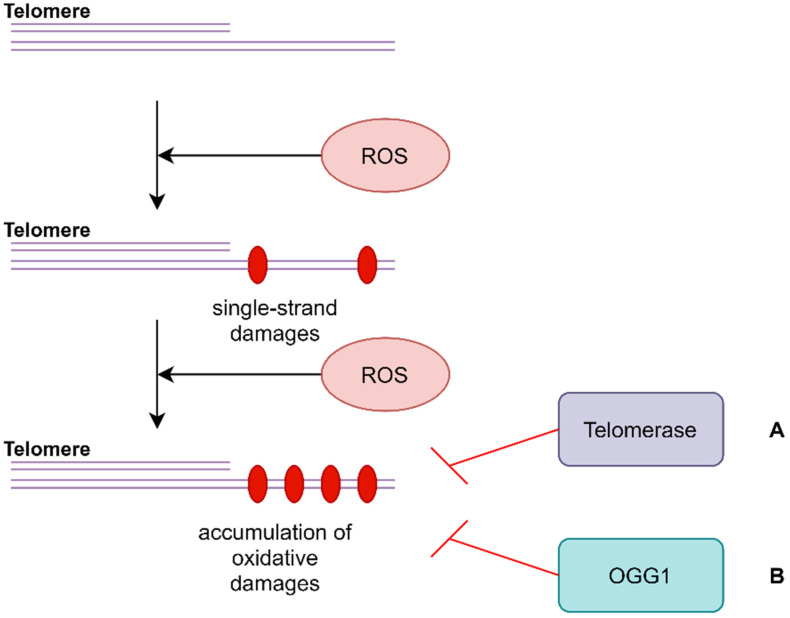
Oxidative telomere damage or single-strand telomere damage prevents the binding of telomerase and OGG1. Accumulation of oxidative damage on single-strand fragments of telomeres causes a conformation change of the end of chromosome so it cannot be recognized by telomerase (**A**). OGG1 cannot repair damage on single strand fragments of telomere because it does not have any template for DNA synthesis (**B**). OGG1–8–oxoguanine DNA glycosylase; ROS–reactive oxygen species.

ROS can react with DNA, causing oxidative damage [36] (Figure 1). Telomeres, which are repeats of the 5′-TTAGGG-3′ sequence, are sensitive to guanine oxidation in cases of increased iron binding to these sequences [37]. The accumulation of 8-oxoG at the end of chromosomes due to long exposure to ROS causes a high frequency of single-strand break formation in the DNA backbone. Moreover, oxidative damage to the end of chromosomes may inhibit telomerase activity. The oligonucleotide ending in 8-oxoG (5′-TTA-8oxoG-3′) cannot be extended by a telomerase [38] (Figure 1A). It has been shown that urinary 8-hydroxy-2-deoxyguanosine (a marker of genomic instability) is increased in patients with OSA [39], which supports the validity of the hypothesis.

Barns et al. indicated a possible role of the disturbance of DNA repair mechanisms in telomere attrition–8-oxoguanine DNA glycosylase (OGG1), which is stimulated by APE1 (apurinic/apyrimidinic endodeoxyribonuclease (1), XPC (DNA damage recognition and repair factor) and NEIL1 (Nei like DNA glycosylase 1), activity of MTH1 (nudix hydrolase 1) and the abase excision repair (BER) pathway [35]. BER is a cellular DNA repair mechanism which recognizes DNA damage, excises fragment of DNA, and then inserts the correct sequence [40]. BER can only repair dsDNA fragments, so damage to single-strand fragments of telomeres cannot be repaired (Figure 1B). OGG1 glycosylase binds only to 8-oxoG in front of C, and then removes a pair of bases, but it is not able to repair oxidative damage to ssDNA [35,40]. MTH1 hydrolyzes oxidized dNTPs before they can be incorporated into the genome by DNA polymerases during replication [35]. Interestingly, acute MTH1 depletion increases telomere shortening in cancer cells [41].

None of these pathways and mechanisms were studied in biological material from OSA patients. The possibility that they influence LTL shortening in OSA is only a hypothesis. The available data-evidenced relationship between ROS and telomeric instability is very likely to be present in OSA patients since oxidative stress is one of the key pathophysiological mechanisms of the disorder. Nevertheless, there is a need for studies of this phenomenon in this particular group of patients to verify this hypothesis.

### 4.2. Inflammation and Telomere Length

**Figure 2:**
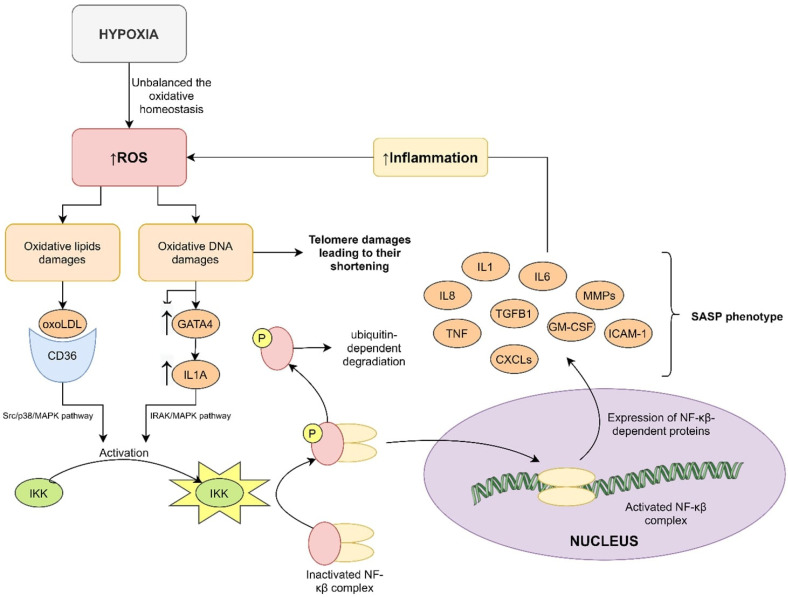
Molecular pathways of telomere shortening and SASP expression. Intermittent hypoxia generates a increased amount (↑) of ROS and develops oxidative stress. ROS damages many cellular components such as lipids or DNA, which activate the NF-κβ complex. NF-κβ is translocated to the nucleus where its function is the translation of many genes including SASP phenotypes. Oxidative DNA damage causes telomere shortening directly by interference with the DNA structure. CD36—scavenger receptor; CXCLs—C-X-C motif chemokine ligands; GATA4—GATA binding protein 4; GM-CSF—granulocyte-macrophage colony-stimulating factor; ICAM-1—intercellular adhesion molecule 1; IKK—kinase of Iκβ; IL1—interleukin 1; IL6—interleukin 6; IL8—interleukin 8; MMPs—metalloproteinases; NF-κβ—nuclear factor κβ; oxoLDL—oxidative law-density lipoprotein; ROS—reactive oxygen species; SASP—senescence associated secretory phenotype; TGFB1—transforming growth factor β1; TNF—tumor necrosis factor; MMPs—metalloproteinases; GM-CSF—granulocyte-macrophage colony-stimulating factor.

The destructive features of ROS are the cause of inflammation in patients with OSA [42] including DNA and lipid damage. Low-density lipoprotein (LDL) oxidates to oxLDL, which is known to be involved in atherosclerosis development. The oxLDL binds to the CD36 on the leukocyte surface and, by the Src/p38/MAPK pathway, leads to activation of the Iκβ kinase (IKK). The phosphorylation of the inhibitor κβ (Iκβ) causes its deactivation and dissociation from the nuclear factor-κβ (NF-κβ) complex, which allows NF-κβ translocation to the nucleus, where can act as an active transcription factor. DNA damage (such as oxoG) has a similar effect on NF-κβ activation, but uses another pathway. DNA damage leads to overexpression of GATA binding protein 4 (GATA4) and interleukin (IL) 1α. IL-1α binds to specific receptors and activates NF-κβ by the IRAK/MAPK pathway [43] (Figure 2).

NF-κβ targets many genes whose protein products include the senescence associated secretory phenotype (SASP) elements–IL-1, IL-6, IL-8, tumor necrosis factor α, transforming growth factor β1, metalloproteinases, C-X-C motif chemokine ligands, granulocyte-macrophage colony-stimulating factor and intercellular adhesion molecule 1 [44]. All these factors have immunomodulatory effects, and they lead to overproduction of ROS in a chronic inflammation mechanism, which works as positive feedback loop [45,46,47]. Studies have confirmed this mechanism in OSA patients [48] but not in terms of LTL.

### 4.3. Hypoxia and Telomere Length

Intermittent hypoxia has also direct impact on LTL in addition to its impact through oxidative stress and inflammation. A factor that mediates hypoxia and hypoxia-dependent response is hypoxia inducible factor 1 (HIF-1), a heterodimer composed of subunits α and β. HIF-1α is the oxygen-dependent subunit which is stabilized during hypoxia or degraded during normoxia [49]. HIF-1 exhibits increased activity in OSA patients [50,51,52,53]. HIF-1 does double duty as a PER-ARNT-SIM (PAS) transcription factor and influences many processes in its targets [54]. One of them is telomerase reverse transcriptase (TERT), which is the catalytic unit of telomerase.

It is hard to draw conclusions from the literature because no research of TERT in OSA patients has been performed. The available knowledge from various studies of cancer and other cell cultures suggests that overactivation of HIF-1 leads to overexpression of TERT, increased level of telomerase in cells and telomere stabilization [55,56,57,58,59]. Hence, it should be expected to find increased expression levels of TERT in OSA patients. However, the aforementioned results mostly show LTL shortening. One possible reason for this inconsistency can be oxidative stress. Intermittent hypoxia in OSA generates much more ROS than sustained hypoxia (e.g., in cancers) [60]. It affects DNA, leading to damage, and disturbs the T-loop structure of telomeres. Conformation change prevents telomerase binding and the regeneration of telomeres [38]. These mechanisms can also explain LTL lengthening in childhood OSA [19]; the DNA damage in these cases is not as advanced as in adulthood, which is probably enabled by telomerase binding. There is also evidence of the influence of TERT on the increased activation of NF-κβ by the stepped-up translocation of this protein to the nucleus [61].

## 5. Circadian Clock Disturbances and Accelerated Aging

The frequent arousals present in OSA are responsible for circadian clock disturbances, making them an integral part of the disorder. The human molecular circadian clock is based on a transcriptional negative feedback loop between activators and repressors [53,62]. Among the activators brain and muscle ARNT-like 1 (BMAL1) and clock circadian regulator (CLOCK) [63], which bind together, are worth mention as they create an active transcription complex [64]. BMAL1:CLOCK recognizes E-box motifs (5′CACGTC-3′) in promotors of targeted genes, including repressors, and leads to their transcription, including period proteins (PER) and cryptochromes (CRY) [65,66]. Next, the repressor proteins heterodimerize in the cytoplasm. PER:CRY undergoes phosphorylation due to casein kinases (CKIδ and CKIε) and translocation to the nucleus, where the complex can act as an inhibitor of BMAL1:CLOCK-dependent transcription [67,68]. The cytoplasm and nucleus levels of circadian clock repressors are regulated by the E3 ubiquitin ligase complex (SCF-Fbxl3 complex) and the proteasome-dependent pathways of protein degradation [69,70,71,72]. In investigation of the relationship between the circadian clock and hypoxia in OSA patients, positive correlations between evening PER1, CRY1, and CLOCK proteins and evening HIF-1α protein levels have been found [73], which offer a possible molecular mechanism of circadian clock disturbance in OSA.

It is commonly known that aging changes circadian behavior and metabolism, as has been clearly described [74]. Presumably, the basis of this process is the disruption of the molecular mechanism of circadian clocks and changes in the neuroendocrine activity of the suprachiasmatic nucleus. Senescence impairs clock gene expression in vivo and in vitro through reduced BMAL1 and PER2 expression [75]. Knockout of BMAL1 can cause sarcopenia, cataracts, slowed hair growth and a significantly shorter lifespan in mice [76]. Similar outcomes of lifespan were obtained in Drosophila [77]. Park et al. found that BMAL1 is associated with telomere length in zebrafish as well as mice; it is characterized by rhythmical binding to the ends of chromosomes, which may lead to the conclusion that the circadian clock components are greatly involved in telomere homeostasis regulation [78]. Moreover, the rhythms in the heterochromatin at the telomeres in zebrafish and mice support this hypothesis [78]. Furthermore, BMAL1 probably takes part in the rhythmical epigenetics of telomeres and regulates the expression of a long non-coding RNA called telomeric repeat-containing RNA (TERRA), which is implicated in telomere protection by the recruitment of epigenetic factors [78]. BMAL1^-/*-*^ mice are characterized by premature aging in cases of increased ROS production and cardiac telomere shortening. However, antioxidant therapy improved telomere length and oxidative telomere damages [79]. A study by Khapre et al. showed increased amount of senescence cells in different tissues in BMAL1^−/−^ mice and increased sensitivity to oxidative stress [80]. Another study was conducted on the senescence of hematopoietic progenitor cells (HPC) in patients infected by HIV, whose outcomes showed that the low proliferative rate of HPC was correlated with increased PER2 expression. Furthermore, SIRT1, a negative regulator of PER2, was downregulated. Interestingly, after treatment with resveratrol, which is a phenol compound possessing antioxidant properties, SIRT1 levels increased and PER2 expression decreased [81]. Additionally, Chen et al. verified that telomerase activity and TERT expression oscillate with an endogenous circadian rhythm. What is more, they also found CLOCK^−/−^ mice were characterized by opposite outcomes and telomere shortening [82]. In the case of the presence of a E-box motif in the TERT promotor, they also confirmed that it as one of BMAL1:CLOCK complex targets [82].

## 6. Conclusions

The molecular mechanisms connecting OSA and accelerated aging are very complicated. They are based on oxidative stress, which is dependent on intermittent hypoxia and inflammation. These mechanisms create a positive feedback loop which accelerates oxidative telomere damage and leads to premature cellular senescence. Additionally, circadian clock disruption most likely also takes part in this process.

Given the increased prevalence of OSA and its age-related complications, it seems to be important to explore the pathomechanism of cellular senescence and, especially in terms of the clinical use of antioxidant and CPAP treatments, monitoring their effectiveness and assessing the occurrence of OSA complications.

## Abbreviations

AHI—apnea-hypopnea index; BER—base excision repair; BMAL1—brain and muscle ARNT-like 1; BMI—body mass index; CD36—CD36 molecule; CKIδ—casein kinase I isoform delta; CKIε—casein kinase I isoform epsilon; CLOCK—clock circadian regulator; CPAP—continuous-positive airway pressure; CRY—cryptochromes; CXCLs—C-X-C motif chemokine ligands; DNA—deoxyribonucleic acid; dNTPs—deoxynucleotide triphosphates; dsDNA—double strand DNA; GATA4—GATA binding protein 4; GM-CSF—granulocyte-macrophage colony-stimulating factor; HIF-1—hypoxia inducible factor-1; HIV—human immunodeficiency virus; HPC—hematopoietic progenitor cells; ICAM-1—intercellular adhesion molecule 1; Iκβ—inhibitor of NF-κβ; IKK—kinase of Iκβ; IL-1—interleukin 1; IL-6—interleukin 6; IL-8—interleukin 8; IRAK—interleukin 1 receptor-associated kinase 1; LDL—low density lipoprotein; LTL—leukocyte telomere length; MAPK—mitogen-activated protein kinase 1; MMPs—metalloproteinases; MTH1—nudix hydrolase 1; NEIL1—Nei like DNA glycosylase 1; NF-κβ—nuclear factor kappa-beta; ODI—oxygen desaturation index; OGG1—8-oxoguanine DNA glycosylase; OSA—obstructive sleep apnea; oxLDL—oxidative LDL; p38—mitogen-activated protein kinase 14; PAS domain—PER-ARNT-SIM domain; PSQI—Pittsburgh Sleep Quality Index; RDI—respiratory disturbance index; ROS—reactive oxygen species; SASP—senescence-associated secretory phenotype; SIRT1—sirtuin 1; Src—SRC proto-oncogene; ssDNA—single strand DNA; TERRA—telomeric repeat-containing RNA; TERT—telomerase reverse transcriptase; TGF-β1—transforming growth factor beta 1; TNF—tumor necrosis factor; XPC—DNA damage recognition and repair factor.

## References

[1] Lévy P., Kohler M., McNicholas W.T., Barbé F., McEvoy R.D., Somers V.K., Lavie L., Pépin J.L. (2015). Obstructive sleep apnoea syndrome. Nat. Rev. Dis. Primers.

[2] Mokros Ł., Kuczynski W., Gabryelska A., Franczak Ł., Spałka J., Białasiewicz P. (2018). High Negative Predictive Value of Normal Body Mass Index for Obstructive Sleep Apnea in the Lateral Sleeping Position. J. Clin. Sleep Med..

[3] Bradley T.D., Floras J.S. (2009). Obstructive sleep apnoea and its cardiovascular consequences. Lancet.

[4] Gabryelska A., Łukasik Z.M., Makowska J.S., Białasiewicz P. (2018). Obstructive Sleep Apnea: From Intermittent Hypoxia to Cardiovascular Complications via Blood Platelets. Front. Neurol..

[5] Gabryelska A., Karuga F.F., Szmyd B., Białasiewicz P. (2020). HIF-1α as a Mediator of Insulin Resistance, T2DM, and Its Complications: Potential Links with Obstructive Sleep Apnea. Front. Physiol..

[6] Gabryelska A., Chrzanowski J., Sochal M., Kaczmarski P., Turkiewicz S., Ditmer M., Karuga F.F., Czupryniak L., Białasiewicz P. (2021). Nocturnal Oxygen Saturation Parameters as Independent Risk Factors for Type 2 Diabetes Mellitus among Obstructive Sleep Apnea Patients. J. Clin. Med..

[7] Hunyor I., Cook K.M. (2018). Models of intermittent hypoxia and obstructive sleep apnea: Molecular pathways and their contribution to cancer. Am. J. Physiol. Regul. Integr. Comp. Physiol..

[8] Wei W., Ji S. (2018). Cellular senescence: Molecular mechanisms and pathogenicity. J. Cell. Physiol..

[9] Noren Hooten N., Evans M.K. (2017). Techniques to Induce and Quantify Cellular Senescence. J. Vis. Exp..

[10] Hernandez-Segura A., Nehme J., Demaria M. (2018). Hallmarks of Cellular Senescence. Trends Cell. Biol..

[11] Childs B.G., Durik M., Baker D.J., van Deursen J.M. (2015). Cellular senescence in aging and age-related disease: From mechanisms to therapy. Nat. Med..

[12] Turner K.J., Vasu V., Griffin D.K. (2019). Telomere Biology and Human Phenotype. Cells.

[13] Mander B.A., Winer J.R., Walker M.P. (2017). Sleep and Human Aging. Neuron.

[14] Prather A.A., Puterman E., Lin J., O’Donovan A., Krauss J., Tomiyama A.J., Epel E.S., Blackburn E.H. (2011). Shorter leukocyte telomere length in midlife women with poor sleep quality. J. Aging Res..

[15] Liang G., Schernhammer E., Qi L., Gao X., De Vivo I., Han J. (2011). Associations between rotating night shifts, sleep duration, and telomere length in women. PLoS ONE.

[16] Cribbet M.R., Carlisle M., Cawthon R.M., Uchino B.N., Williams P.G., Smith T.W., Gunn H.E., Light K.C. (2014). Cellular aging and restorative processes: Subjective sleep quality and duration moderate the association between age and telomere length in a sample of middle-aged and older adults. Sleep.

[17] Jackowska M., Hamer M., Carvalho L.A., Erusalimsky J.D., Butcher L., Steptoe A. (2012). Short sleep duration is associated with shorter telomere length in healthy men: Findings from the Whitehall II cohort study. PLoS ONE.

[18] Garland S.N., Palmer C., Donelson M., Gehrman P., Johnson F.B., Mao J.J. (2014). A nested case-controlled comparison of telomere length and psychological functioning in breast cancer survivors with and without insomnia symptoms. Rejuvenation Res..

[19] Kim J., Lee S., Bhattacharjee R., Khalyfa A., Kheirandish-Gozal L., Gozal D. (2010). Leukocyte telomere length and plasma catestatin and myeloid-related protein 8/14 concentrations in children with obstructive sleep apnea. Chest.

[20] Polonis K., Sompalli S., Becari C., Xie J., Covassin N., Schulte P.J., Druliner B.R., Johnson R.A., Narkiewicz K., Boardman L.A. (2019). Telomere Length and Risk of Major Adverse Cardiac Events and Cancer in Obstructive Sleep Apnea Patients. Cells.

[21] Polonis K., Somers V.K., Becari C., Covassin N., Schulte P.J., Druliner B.R., Johnson R.A., Narkiewicz K., Boardman L.A., Singh P. (2017). Moderate-to-severe obstructive sleep apnea is associated with telomere lengthening. Am. J. Physiol. Heart Circ. Physiol..

[22] Barceló A., Piérola J., López-Escribano H., de la Peña M., Soriano J.B., Alonso-Fernández A., Ladaria A., Agustí A. (2010). Telomere shortening in sleep apnea syndrome. Respir. Med..

[23] Savolainen K., Eriksson J.G., Kajantie E., Lahti M., Räikkönen K. (2014). The history of sleep apnea is associated with shorter leukocyte telomere length: The Helsinki Birth Cohort Study. Sleep Med..

[24] Kwon A.M., Baik I., Thomas R.J., Shin C. (2015). The association between leukocyte telomere lengths and sleep instability based on cardiopulmonary coupling analysis. Sleep Breath..

[25] Boyer L., Audureau E., Margarit L., Marcos E., Bizard E., Le Corvoisier P., Macquin-Mavier I., Derumeaux G., Damy T., Drouot X. (2016). Telomere Shortening in Middle-Aged Men with Sleep-disordered Breathing. Ann. Am. Thorac. Soc..

[26] Kim K.S., Kwak J.W., Lim S.J., Park Y.K., Yang H.S., Kim H.J. (2016). Oxidative Stress-induced Telomere Length Shortening of Circulating Leukocyte in Patients with Obstructive Sleep Apnea. Aging Dis..

[27] Choi K.M., Thomas R.J., Yoon D.W., Lee S.K., Baik I., Shin C. (2016). Interaction between Obstructive Sleep Apnea and Shortened Telomere Length on Brain White Matter Abnormality. Sleep.

[28] Riestra P., Gebreab S.Y., Xu R., Khan R.J., Quarels R., Gibbons G., Davis S.K. (2017). Obstructive sleep apnea risk and leukocyte telomere length in African Americans from the MH-GRID study. Sleep Breath..

[29] Tempaku P.F., Mazzotti D.R., Hirotsu C., Andersen M.L., Xavier G., Maurya P.K., Rizzo L.B., Brietzke E., Belangero S.I., Bittencourt L. (2016). The effect of the severity of obstructive sleep apnea syndrome on telomere length. Oncotarget.

[30] Carroll J.E., Irwin M.R., Seeman T.E., Diez-Roux A.V., Prather A.A., Olmstead R., Epel J., Lin R., Redline S. (2019). Obstructive sleep apnea, nighttime arousals, and leukocyte telomere length: The Multi-Ethnic Study of Atherosclerosis. Sleep.

[31] Pinilla L., Santamaria-Martos F., Benítez I.D., Zapater A., Targa A., Mediano O., Masa J.F., Masdeu M.J., Minguez O., Aguilà M. (2021). Association of Obstructive Sleep Apnea with the Aging Process. Ann. Am. Thorac. Soc..

[32] Lin C.C., Wang H.Y., Liaw S.F., Chiu C.H., Lin M.W. (2019). Effect of oral appliance on circulating leukocyte telomere length and SIRT1 in obstructive sleep apnea. Clin. Oral Investig..

[33] Khalyfa A., Marin J.M., Qiao Z., Rubio D.S., Kheirandish-Gozal L., Gozal D. (2020). Plasma exosomes in OSA patients promote endothelial senescence: Effect of long-term adherent continuous positive airway pressure. Sleep.

[34] Wang Y., Zhang S.X., Gozal D. (2010). Reactive oxygen species and the brain in sleep apnea. Respir. Physiol. Neurobiol..

[35] Barnes R.P., Fouquerel E., Opresko P.L. (2019). The impact of oxidative DNA damage and stress on telomere homeostasis. Mech. Ageing Dev..

[36] Cadet J., Wagner J.R. (2013). DNA base damage by reactive oxygen species, oxidizing agents, and UV radiation. Cold Spring Harb. Perspect. Biol..

[37] Oikawa S., Tada-Oikawa S., Kawanishi S. (2001). Site-specific DNA damage at the GGG sequence by UVA involves acceleration of telomere shortening. Biochemistry.

[38] Ahmed W., Lingner J. (2018). Impact of oxidative stress on telomere biology. Differentiation.

[39] Xie J., Jiang J., Shi K., Zhang T., Zhu T., Chen H., Chen R., Qi L., Ding W., Yi Q. (2014). DNA damage in peripheral blood lymphocytes from patients with OSAHS. Sleep Breath..

[40] Iyama T., Wilson D.M. (2013). DNA repair mechanisms in dividing and non-dividing cells. DNA Repair.

[41] Lee M., Hills M., Conomos D., Stutz M.D., Dagg R.A., Lau L.M., Reddel R.R., Pickett H.A. (2014). Telomere extension by telomerase and ALT generates variant repeats by mechanistically distinct processes. Nucleic Acids Res..

[42] de Lima F.F., Mazzotti D.R., Tufik S., Bittencourt L. (2016). The role inflammatory response genes in obstructive sleep apnea syndrome: A review. Sleep Breath..

[43] Weber A., Wasiliew P., Kracht M. (2010). Interleukin-1 (IL-1) pathway. Sci. Signal..

[44] Lopes-Paciencia S., Saint-Germain E., Rowell M.C., Ruiz A.F., Kalegari P., Ferbeyre G. (2019). The senescence-associated secretory phenotype and its regulation. Cytokine.

[45] Brasier A.R. (2006). The NF-kappaB regulatory network. Cardiovasc. Toxicol..

[46] Saretzki G., Harris J., Korolchuk V. (2018). Telomeres, Telomerase and Ageing. Biochemistry and Cell Biology of Ageing: Part I Biomedical Science.

[47] Zhang J., Rane G., Dai X., Shanmugam M.K., Arfuso F., Samy R.P., Lai M.K., Kappei D., Kumar A.P., Sethi G. (2016). Ageing and the telomere connection: An intimate relationship with inflammation. Ageing Res. Rev..

[48] Imamura T., Poulsen O., Haddad G.G. (2016). Intermittent hypoxia induces murine macrophage foam cell formation by IKK-β-dependent NF-κB pathway activation. J. Appl. Physiol..

[49] Ke Q., Costa M. (2006). Hypoxia-inducible factor-1 (HIF-1). Mol. Pharmacol..

[50] Prabhakar N.R., Peng Y.J., Nanduri J. (2020). Hypoxia-inducible factors and obstructive sleep apnea. J. Clin. Investig..

[51] Gabryelska A., Szmyd B., Szemraj J., Stawski R., Sochal M., Białasiewicz P. (2020). Patients with obstructive sleep apnea present with chronic upregulation of serum HIF-1α protein. J. Clin. Sleep Med..

[52] Gabryelska A., Stawski R., Sochal M., Szmyd B., Białasiewicz P. (2020). Influence of one-night CPAP therapy on the changes of HIF-1α protein in OSA patients: A pilot study. J. Sleep Res..

[53] Gabryelska A., Szmyd B., Panek M., Szemraj J., Kuna P., Białasiewicz P. (2020). Serum hypoxia-inducible factor-1α protein level as a diagnostic marker of obstructive sleep apnea. Pol. Arch. Intern. Med..

[54] Wenger R.H., Stiehl D.P., Camenisch G. (2005). Integration of oxygen signaling at the consensus HRE. Sci. STKE.

[55] Song H., Chen X., Jiao Q., Qiu Z., Shen C., Zhang G., Sun Z., Zhang H., Luo Q.Y. (2021). HIF-1α-Mediated Telomerase Reverse Transcriptase Activation Inducing Autophagy Through Mammalian Target of Rapamycin Promotes Papillary Thyroid Carcinoma Progression During Hypoxia Stress. Thyroid.

[56] Yatabe N., Kyo S., Maida Y., Nishi H., Nakamura M., Kanaya T., Tanaka M., Isaka K., Ogawa S., Inoue M. (2004). HIF-1-mediated activation of telomerase in cervical cancer cells. Oncogene.

[57] Lou F., Chen X., Jalink M., Zhu Q., Ge N., Zhao S., Fang X., Fan Y., Björkholm M., Liu Z. (2007). The opposing effect of hypoxia-inducible factor-2alpha on expression of telomerase reverse transcriptase. Mol. Cancer Res..

[58] Nishi H., Nakada T., Kyo S., Inoue M., Shay J.W., Isaka K. (2004). Hypoxia-inducible factor 1 mediates upregulation of telomerase (hTERT). Mol. Cell. Biol..

[59] Cataldi A., Zara S., Rapino M., Zingariello M., di Giacomo V., Antonucci A. (2009). p53 and telomerase control rat myocardial tissue response to hypoxia and ageing. Eur. J. Histochem..

[60] Yeo E.J. (2019). Hypoxia and aging. Exp. Mol. Med..

[61] Liu Q., Sun Y., Lv Y., Le Z., Xin Y., Zhang P., Liu Y. (2016). TERT alleviates irradiation-induced late rectal injury by reducing hypoxia-induced ROS levels through the activation of NF-κB and autophagy. Int. J. Mol. Med..

[62] Sato T.K., Yamada R.G., Ukai H., Baggs J.E., Miraglia L.J., Kobayashi T.J., Welsh D.K., Kay S.A., Ueda H.R., Hogenesch J.B. (2006). Feedback repression is required for mammalian circadian clock function. Nat. Genet..

[63] Siepka S.M., Yoo S.H., Park J., Lee C., Takahashi J.S. (2007). Genetics and neurobiology of circadian clocks in mammals. Cold Spring Harb. Symp. Quant. Biol..

[64] Huang N., Chelliah Y., Shan Y., Taylor C.A., Yoo S.H., Partch C., Green C.B., Zhang H., Takahashi J.S. (2012). Crystal structure of the heterodimeric CLOCK:BMAL1 transcriptional activator complex. Science.

[65] Gallego M., Virshup D.M. (2007). Post-translational modifications regulate the ticking of the circadian clock. Nat. Rev. Mol. Cell. Biol..

[66] Freeman S.L., Kwon H., Portolano N., Parkin G., Venkatraman Girija U., Basran J., Fielding A.J., Fairall L., Svistunenko D.A., Moody P.C. (2019). Heme binding to human CLOCK affects interactions with the E-box. Proc. Natl. Acad. Sci. USA.

[67] Partch C.L., Green C.B., Takahashi J.S. (2014). Molecular architecture of the mammalian circadian clock. Trends Cell. Biol..

[68] Welsh D.K., Takahashi J.S., Kay S.A. (2010). Suprachiasmatic nucleus: Cell autonomy and network properties. Annu. Rev. Physiol..

[69] Yoo S.H., Mohawk J.A., Siepka S.M., Shan Y., Huh S.K., Hong H.K., Kornblum I., Vivek K., Koike N., Nussbaum J. (2013). Competing E3 ubiquitin ligases govern circadian periodicity by degradation of CRY in nucleus and cytoplasm. Cell.

[70] Reischl S., Vanselow K., Westermark P.O., Thierfelder N., Maier B., Herzel H., Kramer A. (2007). Beta-TrCP1-mediated degradation of PERIOD2 is essential for circadian dynamics. J. Biol. Rhythms..

[71] Busino L., Bassermann F., Maiolica A., Lee C., Nolan P.M., Godinho S.I., Draetta G.F., Pagano M. (2007). SCFFbxl3 controls the oscillation of the circadian clock by directing the degradation of cryptochrome proteins. Science.

[72] Siepka S.M., Yoo S.H., Park J., Song W., Kumar V., Hu Y., Lee C., Takahashi J.S. (2007). Circadian mutant Overtime reveals F-box protein FBXL3 regulation of cryptochrome and period gene expression. Cell.

[73] Gabryelska A., Sochal M., Turkiewicz S., Białasiewicz P. (2020). Relationship between HIF-1 and Circadian Clock Proteins in Obstructive Sleep Apnea Patients-Preliminary Study. J. Clin. Med..

[74] Hood S., Amir S. (2017). The aging clock: Circadian rhythms and later life. J. Clin. Investig..

[75] Kunieda T., Minamino T., Katsuno T., Tateno K., Nishi J., Miyauchi H., Orimo M., Oskada S., Komuro I. (2006). Cellular senescence impairs circadian expression of clock genes in vitro and in vivo. Circ. Res..

[76] Kondratov R.V., Kondratova A.A., Gorbacheva V.Y., Vykhovanets O.V., Antoch M.P. (2006). Early aging and age-related pathologies in mice deficient in BMAL1, the core componentof the circadian clock. Genes Dev..

[77] Krishnan N., Kretzschmar D., Rakshit K., Chow E., Giebultowicz J.M. (2009). The circadian clock gene period extends healthspan in aging *Drosophila melanogaster*. Aging.

[78] Park J., Zhu Q., Mirek E., Na L., Raduwan H., Anthony T.G., Belden W.J. (2019). BMAL1 associates with chromosome ends to control rhythms in TERRA and telomeric heterochromatin. PLoS ONE.

[79] Hemmeryckx B., Hohensinner P., Swinnen M., Heggermont W., Wojta J., Lijnen H.R. (2016). Antioxidant Treatment Improves Cardiac Dysfunction in a Murine Model of Premature Aging. J. Cardiovasc. Pharmacol..

[80] Khapre R.V., Kondratova A.A., Susova O., Kondratov R.V. (2011). Circadian clock protein BMAL1 regulates cellular senescence in vivo. Cell Cycle.

[81] Bordoni V., Tartaglia E., Refolo G., Sacchi A., Grassi G., Antinori A., Fimia G.M., Agrati C. (2020). Per2 Upregulation in Circulating Hematopoietic Progenitor Cells During Chronic HIV Infection. Front. Cell. Infect. Microbiol..

[82] Chen W.D., Wen M.S., Shie S.S., Lo Y.L., Wo H.T., Wang C.C., Hsieh I.C., Lee T.H., Wang C.Y. (2014). The circadian rhythm controls telomeres and telomerase activity. Biochem. Biophys. Res. Commun..

